# Spatial prediction and validation of zoonotic hazard through micro-habitat properties: where does Puumala hantavirus hole – up?

**DOI:** 10.1186/s12879-017-2618-z

**Published:** 2017-07-26

**Authors:** Hussein Khalil, Gert Olsson, Magnus Magnusson, Magnus Evander, Birger Hörnfeldt, Frauke Ecke

**Affiliations:** 1 Department of Wildlife, Fish, and Environmental Studies, Swedish University of Agricultural Sciences, Skogmarksgränd, 901 83 Umeå, Sweden; 2 Department of Clinical Microbiology, Virology, Umeå University, 901 85 Umeå, Sweden; 3 Department of Aquatic Sciences and Assessment, Swedish University of Agricultural Sciences, Gerda Nilssons väg 5, 756 51 Uppsala, Sweden

**Keywords:** Bank vole, Boosted regression trees, Hantavirus, Machine learning, Micro-habitat, Prediction, Puumala virus, Validation, Zoonotic hazard

## Abstract

**Background:**

To predict the risk of infectious diseases originating in wildlife, it is important to identify habitats that allow the co-occurrence of pathogens and their hosts. Puumala hantavirus (PUUV) is a directly-transmitted RNA virus that causes hemorrhagic fever in humans, and is carried and transmitted by the bank vole (*Myodes glareolus*). In northern Sweden, bank voles undergo 3–4 year population cycles, during which their spatial distribution varies greatly.

**Methods:**

We used boosted regression trees; a technique inspired by machine learning, on a 10 – year time-series (fall 2003–2013) to develop a spatial predictive model assessing seasonal PUUV hazard using micro-habitat variables in a landscape heavily modified by forestry. We validated the models in an independent study area approx. 200 km away by predicting seasonal presence of infected bank voles in a five-year-period (2007–2010 and 2015).

**Results:**

The distribution of PUUV-infected voles varied seasonally and inter-annually. In spring, micro-habitat variables related to cover and food availability in forests predicted both bank vole and infected bank vole presence. In fall, the presence of PUUV-infected voles was generally restricted to spruce forests where cover was abundant, despite the broad landscape distribution of bank voles in general. We hypothesize that the discrepancy in distribution between infected and uninfected hosts in fall, was related to higher survival of PUUV and/or PUUV-infected voles in the environment, especially where cover is plentiful.

**Conclusions:**

Moist and mesic old spruce forests, with abundant cover such as large holes and bilberry shrubs, also providing food, were most likely to harbor infected bank voles. The models developed using long-term and spatially extensive data can be extrapolated to other areas in northern Fennoscandia. To predict the hazard of directly transmitted zoonoses in areas with unknown risk status, models based on micro-habitat variables and developed through machine learning techniques in well-studied systems, could be used.

**Electronic supplementary material:**

The online version of this article (doi:10.1186/s12879-017-2618-z) contains supplementary material, which is available to authorized users.

## Background

Zoonotic disease hazard is contingent upon the spatial overlap between pathogens and their hosts and vectors, realized within an environmental envelope shaped by biotic and abiotic factors. The transmission of zoonotic pathogens requires close contact between infected individuals on one hand and vectors or susceptible hosts on the other, and is therefore essentially a spatial phenomenon [1]. The recognition of habitat variables that capacitate pathogen, host, and vector co-occurrence enables the prediction of zoonotic hazard in a world where emerging infectious diseases pose an increasing socio-economic threat [2].

For vector-borne diseases, the distribution of arthropod vectors such as ticks and mosquitos, which transmit important zoonoses such as Lyme disease and West Nile virus, is often climatically delimited. Survival and vectorial capacity of ticks and mosquitoes are affected by factors such as humidity [3] and temperature [4]. Warm-blooded hosts on the other hand are less affected by climatic variables [1]. For example, small mammal hosts of hantaviruses [5–7], arenavirus [8], and plague [9] are often dependent on food, structural habitat, and landscape factors. The density and distribution of some host populations vary considerably between seasons and years, which poses an additional challenge of identifying habitats that serve as ‘refugia’ for a pathogen when its host distribution contracts [10].

The bank vole (*Myodes glareolus*) is the most common small mammal in Europe and has a wide distribution in Europe and Asia [11]. In northern Fennoscandia, bank vole populations undergo 3–4 year cycles [12–14] characterized by large variation in density and landscape distribution. The bank vole is the sole host of Puumala hantavirus (PUUV, genus *Hantavirus*, family *Bunyaviridae*) [15, 16], an RNA virus that causes a mild form of hemorrhagic fever in humans and responsible for thousands of cases each year [17]. PUUV is directly transmitted among bank voles through physical contact, e.g. grooming and biting, or environmentally through inhalation of viral particles excreted in feces or urine. PUUV tracks the dynamic distribution of its host over the course of a population cycle. Infection rates and presence of infected voles vary over few kilometers and from one year to the next [6, 18, 19].

In northern Sweden, the bank vole is the most abundant small mammal [13] and is generally considered a forest dwelling species [20]. The region has been modified by forestry over the last six decades, and approximately 40% of the landscape consists of forests that has been clear-cut at some point [21]. Young even-aged forests lack the extensive three-dimensional structures and ground cover found in older forests [22], which provide shelter and food for forest-dwelling voles [23]. In young forests and clear-cuts, bank vole population densities may reach high levels [23], but over-winter survival of bank voles is highest in old forests [23, 24]. In Western Europe, bank vole density is also highest in habitats with high availability of cover, nesting opportunities, and food [25–27].

Unsurprisingly, PUUV risk is generally associated with forests. Increased logging of old forest reduces the distribution of PUUV-infected voles in the study area; which are more likely to survive winter in old forests [6, 23]. Also, the abundance of cover and food was associated with high fall density of PUUV-infected voles in the same region, however, these results were based on one trapping occasion [28]. In humans, PUUV infection appears more likely in households close to contiguous forests near the coast in northern Sweden [29].

Nevertheless, the occurrence of hantaviruses does not always match that of their hosts, neither at a continental [30] nor regional scales [31], and the causes behind this discrepancy are unclear. Further, little is currently known about the properties of infection “refugia” where the virus persists during periods with low host density. Characterizing these habitats enables mitigating zoonotic risk by managing the relatively few sites from which infection spreads in the landscape when host populations increase. Finally, according to our knowledge, the predictive power and robustness of local habitat models for hantavirus presence remain untested.

Here, we used boosted regression trees, a technique inspired by machine learning, on a 10-year dataset to (a) identify micro-habitat characteristics important for bank vole presence, and more importantly, the presence of infected bank voles in spring and fall. We then (b) validate the models in an independent study area by predicting seasonal presence of infected voles in a five-year-period. Finally, due to the dynamic nature of bank vole and PUUV presence in the landscape, we also (c) seek key habitats where PUUV persists when bank vole densities are low, i.e. infection ‘refugia’. We hypothesize that forests rich in cover and food are important for the presence of infected bank voles [28], both through maintaining bank vole populations and promoting PUUV survival in the environment by providing shade and moisture [32, 33].The predictive framework developed and validated here can be used by practitioners and stakeholders to assess zoonotic PUUV hazard using micro-habitat variables.

## Methods

### Bank vole and infection data

#### Training data

Bank vole data in fall 2003–2013 was available through the Swedish Environmental Monitoring Program [13]. The study area is located in northern Sweden (approx. 64 ° N, 20 ° E) and belongs to the middle boreal zone [34]. Within a total area of 100 × 100 km, small mammals are trapped twice a year – spring (May) and fall (September) – in 58 systematically placed 1-ha plots (see [12, 13, 34] for further details). Each sampling plot contains 10 trapping stations 10 m apart; unless any of the trapping stations fell within non-trappable locations such as lakes. Each plot is trapped for three nights and the total trapping effort is 150 trap nights. We classified the years between fall 2003 and 2013 based on the phases of the vole population cycles as follows: ‘increase’, ‘peak’, and ‘decline’ years [35]. For the four-year cycle between 2009 and 2012, there was an additional ‘low’ phase.

#### Independent validation data

To validate our predictions, we used unpublished trapping data from a project focusing on the response of small mammals to a forest fire. The study was performed 200 km north of the study area were the training data was collected (approx. 66 ° N, 20 ° E). The trapping of small mammals followed the same protocol as that for the training data, including spring and fall trapping. Sampling occurred from spring 2007 to fall 2010 as well as spring and fall 2015 in 17 1-ha plots. The micro-habitat data collected for the independent validation data was a subset of that for the training data (Table 1), but included the variables that were important for predicting presence of infected voles in the training data.

**Table 1:** Micro-habitat variables used to predict the presence of all bank voles and infected bank voles in 58 1-ha plots in fall 2003–2013 and to validate the models in an independent area (+) (17 plots in 2007–2010 and 2015)

Variable	Explanation	Measure	Availability in validation data
Shrubs	Shrub layer 0.5–5 m in height	5 graded scale	+
Lholes	All large holes >5 cm in diameter	6 number classes	+
Stoneholes	Only holes under stones	6 number classes	−
FWD	Fine woody debris <10 cm	5 graded scale	+
CWD	Coarse woody debris	Total length	+
Cobbles	Stones between 10 and 50 cm in diameter	5 graded scale	+
Lcobbles	Stones >50 cm in diameter	5 graded scale	−
Uveg	Vegetation cover >50 cm	5 graded scale	+
Flveg	Vegetation in field layer ≤50 cm	5 graded scale	+
Grasses	Grass cover	5 graded scale	+
Lichens	Cover of ground lichens	5 graded scale	+
Mosses	Cover of ground and stone mosses	5 graded scale	+
Bilberry	Cover of bilberry	5 graded scale	+
Lingon	Cover of lingonberry	5 graded scale	+
Spruce	Cover of spruce trees	%	+
Pine	Cover of pine trees	%	+
Birch	Cover of pine trees	%	+
Tl1	Upper tree layer (≥ 5 m)	5 graded scale	+
Tl2	Lower tree layer (< 5 m)	5 graded scale	+

For the independent validation data, data on large cobbles and stone holes was not available (−). See also Additional file 1 for more detailed description of the variables and their estimation

#### PUUV data

Data on PUUV infection in bank voles was available in fall 2003–2013 (see [36]). We analyzed serum samples from bank voles by enzyme-linked immunosorbent assay (ELISA) to detect anti-PUUV IgG antibodies and thus sero-positive voles (see [37] for details) in 2003–2013. PUUV infection is chronic and infected voles shed the virus for life [38]. Thus sero-positive bank voles were considered infected and referred to as such throughout the paper. However, bank voles weighing <14.4 g may carry maternal antibodies and were consequently excluded from further analysis since their sero-status may not reflect genuine infection [39]. In subsequent analyses, we used presence-absence data on bank voles in general and PUUV-infected bank voles in 58 1-ha plots in fall 2003–2013.

### Micro-habitat data

Field surveys were done in fall 2012 and 2013 and micro-habitat data was collected from all 58 1-ha sampling plots. At each trapping station, the vegetation and structural habitat variables were collected within a quadratic plot with 2.5 m sides centered on each trapping station (see Table 1 for all measured variables and Additional file 1 for the protocol with definition of variables; see also [23, 40]). Surveyed habitat types included old forest dominated by spruce (*Picea abies*) or pine (*Pinus sylvestris*) (> 80 years-old), intermediate aged forest (20–80 years), clear-cuts (0–20 years), mires and meadows [36]. The majority of the sampling plots were located within forested land and all of the forest vegetation types (lichen, moist, mesic and wet forest) were represented (see [41, 42] for definition of forest vegetation types).

### Statistical analyses

We aimed to develop and independently validate a model predicting presence of PUUV infected bank voles. We used boosted regression trees (BRT), a technique inspired by machine learning methods and characterized by strong predictive performance [43, 44]. BRT combines regression trees [45] and boosting, which is a stage-wise procedure for minimizing a loss function such as deviance [46]. One important difference between BRT and traditional statistical techniques, e.g. generalized linear models (GLM), is that BRT does not fit a single best model but combines a large number of regression tree models to minimize predictive error. Hence, the final model consists of hundreds or thousands of single trees that combine to predict the response. BRT is generally superior in predictive power compared to GLMs or generalized additive models (GAMs) [44, 47] and can handle a large number of predictors of any type (numeric, categorical, etc.) with different scales of measurement. Also, BRT is insensitive to outliers and captures non-linear relationships between response and predictors. If complex enough trees are specified, BRT automatically models interactions among predictors. See Elith et al. [44] for a comprehensive guide for the use of BRT in ecological modelling.

BRT models do not provide *P* values. The use (and abuse) of *P* values are a current topic of debate [48] and our aim to predict PUUV hazard makes model performance our priority [49]. Predictors important in a model are those that appear in many of the fitted regression trees and improve the fit. Relative importance of a predictor is based on the number of times a predictor is selected for splitting a tree, weighted by its contribution to the model due to that split, and averaged over all trees. The relative importance (%) of predictors is scaled so that the total sum is 100, with higher values indicating increasing importance [50]. Partial dependence plots help visualize the curvilinear relationship between the response and predictors and are partly presented in the results.

Despite our focus on maximizing the predictive ability to independent data, we do not treat the output of BRT as a black box. We interpreted the general patterns describing overall bank vole and PUUV landscape distribution patterns. Fitted models are a form of logistic regression, modelling the probability of occurrence of any vole or an infected vole (y = 1) at each sampling plot in spring or fall, given a number of predictors (***X***).The probability is modelled using a logit link function: logit *P* (y = 1| ***X***) = *ƒ* (***X***)**.** BRT should be interpreted with caution since the fitted relationships may be noisy [44]. Fitting the same model several times to one data set will result in slightly different outputs due to subsets of the data being drawn stochastically for fitting as the model is developed. We restricted our discussion of predictors in the model to the minimum number of predictors that cumulatively reach relative importance of 85%. Beyond 85%, remaining variables contribute a small percentage each, often 1–2% or less. Nevertheless, the full models were used for validation.

To identify micro-habitat variables important in predicting presence of voles in general or infected voles, we fitted two BRT models for spring and fall (thus four models in total) using the variables listed in Table 1. We excluded two variables from BRT models that were highly correlated with others to reduce redundancy: tree lichens and mosses. Then, we used principal component analysis (PCA) to visualize the sampling plots in environmental space defined by the micro-habitat variables we collected. Through bi-plots of PCAs we also highlighted factors important for predicting the presence of voles in general or of infected voles, elucidating the overlap in predictors between models for the presence of bank voles in general and infected bank voles in each season.

Then, we used the models developed on training data to predict the presence of infected bank voles in spring and fall in the independent area (see above). We used the following measures to evaluate model performance: Area Under Curve (AUC), True Positive Rate (TPR), and True Negative Rate (TNR). AUC can be interpreted as the probability of the model assigning a randomly selected positive instant, i.e. a plot with an infected bank vole, a higher probability than a randomly selected negative instant [51]. TPR, also known as sensitivity, assesses the ability of the model to identify presences. It is measured by dividing the number of correct positive instances predicted by the model by total positive instances, i.e. correctly predicted plus misclassified as negative by the model. TNR, also known as specificity, is the number of negative instances correctly identified by the model divided by total number of negative instances [52].

Further, to show where infected bank voles were frequently present, we calculated the number of years when at least one PUUV-infected bank vole was trapped in each plot in spring and fall in fall 2003–2013.

All statistical analyses were performed in R environment [53], version 3.2.2, and “gbm” package [54].

## Results

We analyzed a total of 4169 bank voles trapped in fall 2003–2013. Overall, 942 voles were PUUV-infected, i.e. prevalence was 22.5%. Total PUUV prevalence was 47% in spring and 17% in fall. Bank vole density was higher and their distribution more extensive in fall following summer reproduction compared to spring. In spring, bank voles and infected bank voles were present in 7–81% and 2–70% of the 58 1-ha plots, respectively. In fall, bank voles were present in 30 to 98% of the plots, whereas infected bank voles were present in 2–74%.

**Fig. 1:**
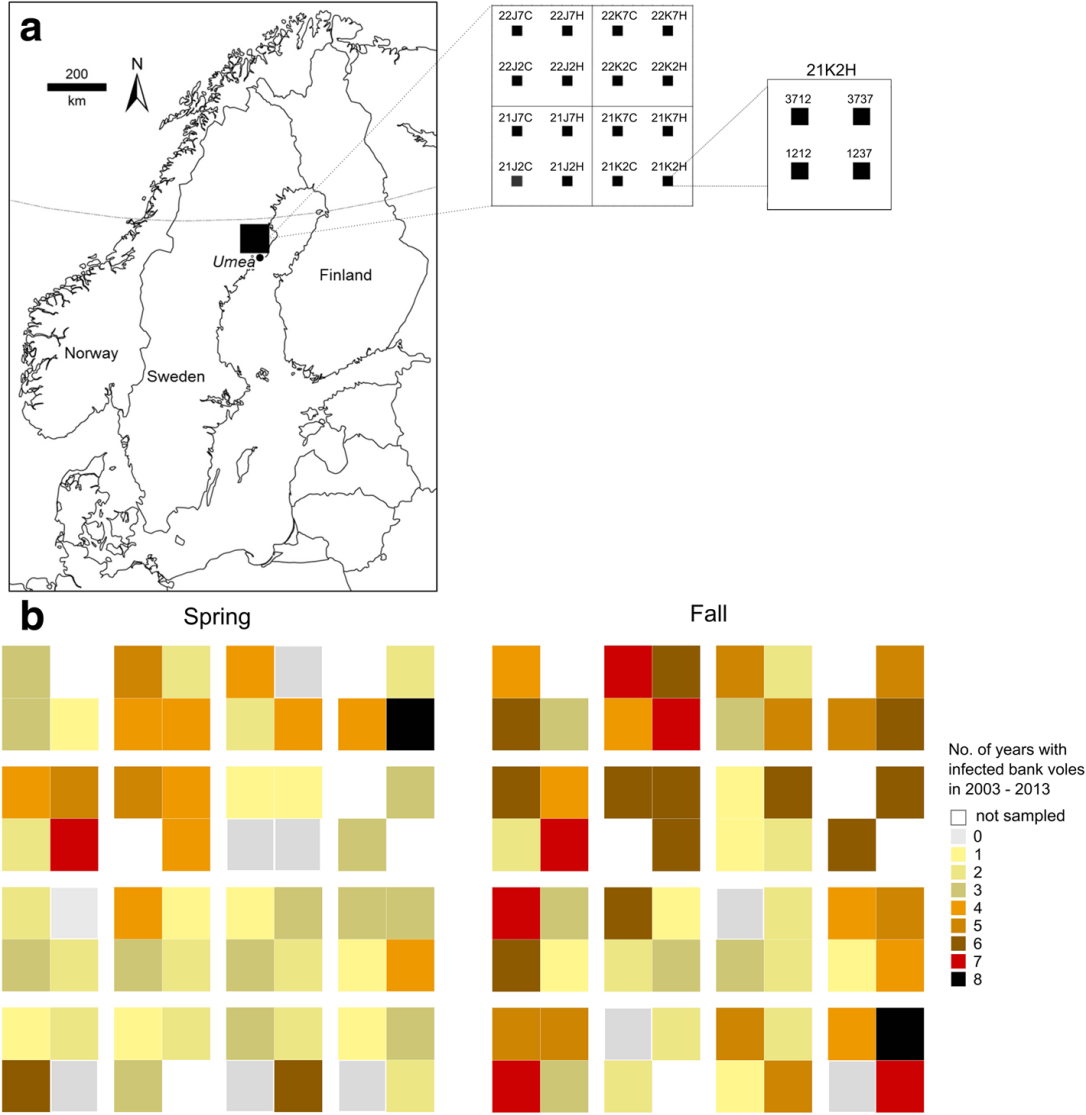
The study area in northern Sweden (*black square*) near the city of Umeå (**a**); the *curved line* indicates 65°N. The blow-up shows the 5 × 5 km landscapes, containing four 1-ha trapping plots each, totalling 58 trappable out of 64 plots (six plots encompassed for example water bodies and were not sampled). **b**) Each tile in the spring and fall panels represents a 1-ha plot and the colour coding reflects the number of years when infected bank voles were trapped, in spring and fall, between fall 2003–2013

**Fig. 2:**
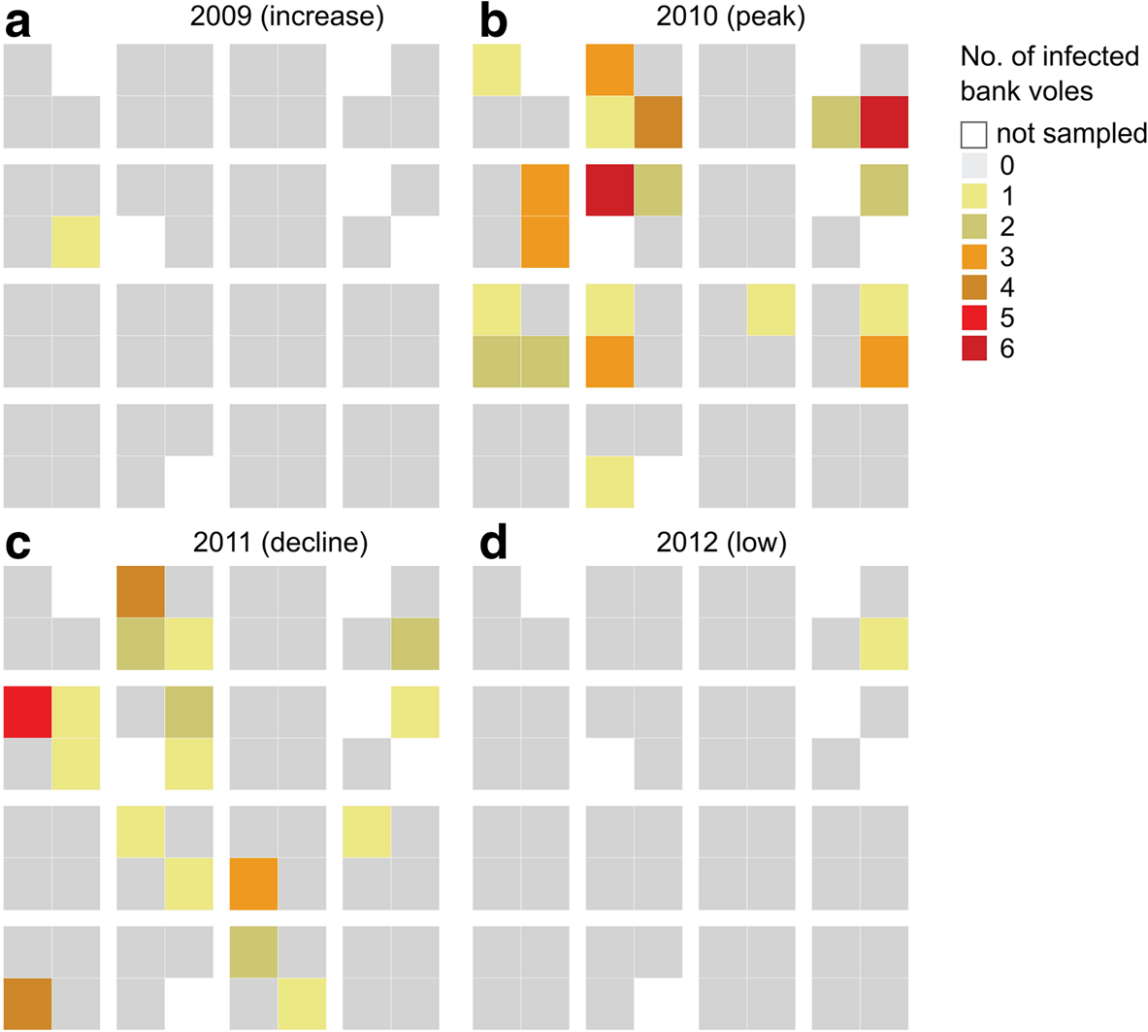
No. of infected bank voles trapped per 100 trap nights in each of the 58 1-ha trapping plots in spring over the course of a complete bank vole population cycle; the only 4-yr. cycle (increase phase to low phase: **a**-**d**: 2009–2012). Six plots encompassed water bodies or other untrappable sites and were not sampled. In 2009 and 2012, infected bank voles were trapped in one plot only

The presence and frequency of occurrence of PUUV-infected voles showed considerable spatial variation. In spring for example, we did not trap infected voles in six out of 58 plots during the study period, whereas in one plot we trapped infected voles on eight occasions out of ten (Fig. 1). There were few plots where PUUV-infected voles were frequently present in spring, when bank vole densities were at an annual low (e.g. Fig. 2, Additional file 2), including four where infected bank voles were trapped on six or more occasions out of ten (Fig. 1).

**Fig. 3:**
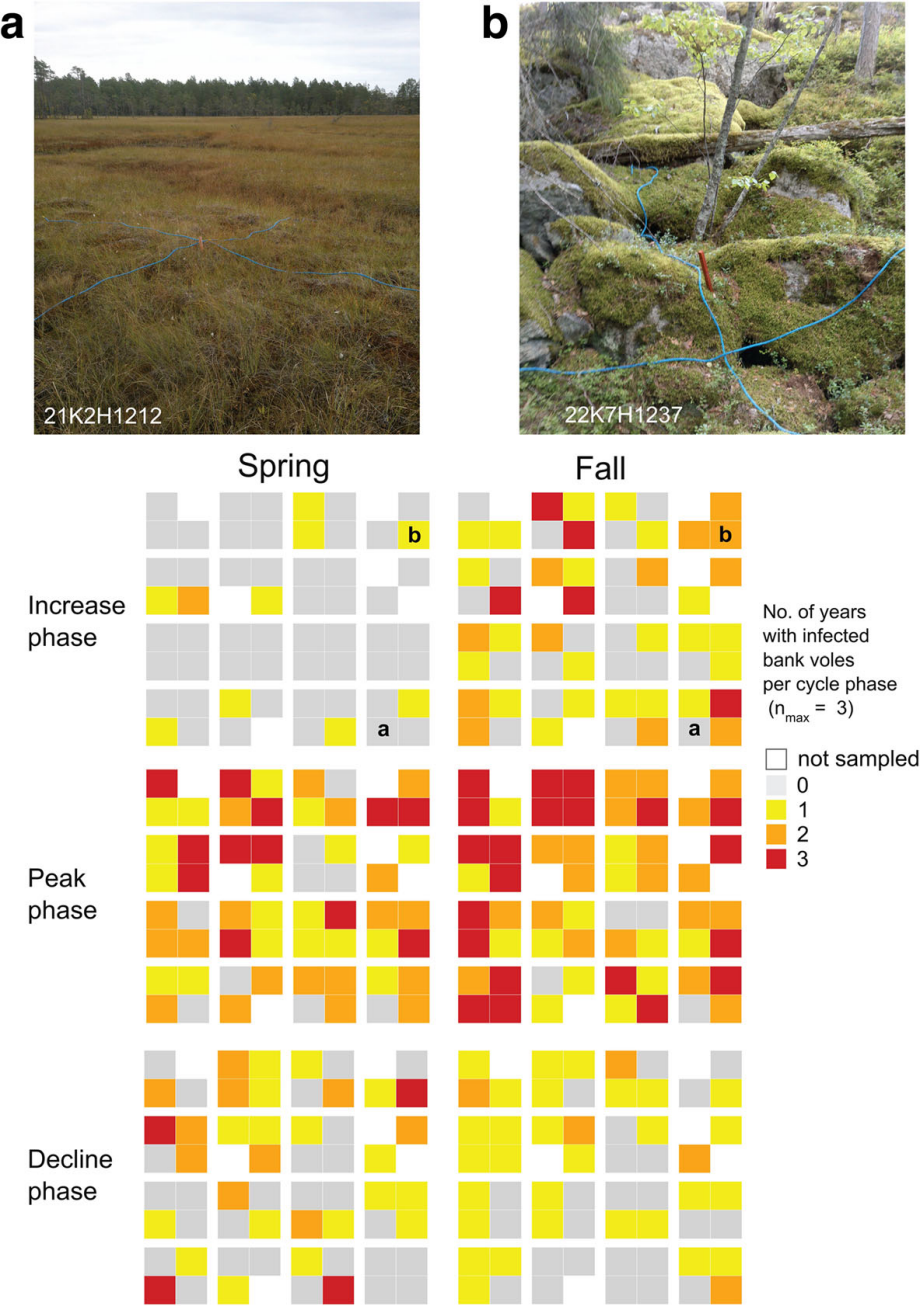
Number of years when infected bank voles were trapped per season and phase of the population cycle. Data were available for three cycles, two 3-year cycles (including fall 2003–fall 2008; fall 2013) and one 4-year cycle (2009–2012). We excluded year 2012, which was the only “low” phase in our study (but see Additional file 2), and thus values ranged between zero and three. The photos (**a** and **b**) show examples of plots where (**a**) infected bank voles were not trapped in either season over the study period, and in (**b**) infected bank voles were frequently trapped. The plot in (**a**) is an open mire and lacks habitat properties related to structure and cover, whereas the plot in (**b**) is rich in large holes. Photo Copyright: Magnus Magnusson

Compared to a previous study on PUUV spatial dynamics between 1979 and 1986 in the same area [6], changes due to succession or forestry altered the infection status of several plots. For example, the old forest plot of 22K7H1237 (see Fig. 1 for the location) became more likely to harbor PUUV-infected bank voles in this study (Fig. 3) compared to 1979–1986 (Figure 1 in [6]). PUUV-infected animals were trapped in 22K7H1237 in eight springs out of 10 in 2003–13 compared to three springs out of seven in springs 1980–1986. Also, plot 21K2C1237 matured between 1986 and 2003 from a forest <20 years-old to an intermediate-aged forest (20–80 years). Infected bank voles were trapped in six springs out of ten between 2003 and 2013, compared to one spring in 1980–1986. Conversely, plot 21J2C3737 was an old forest in 1980 to 1986 but was cut sometime after, and we trapped infected bank voles there twice in spring between 2003 and 2013, compared to four out of seven times between spring 1980 and spring 1986.

Also, there was large inter-annual variation in landscape occupancy of infected bank voles, depending on the phase of the population cycle (Fig. 3, Additional file 2). For example, in the spring of 2007 – a peak year – infected bank voles were present in 38 out of 58 1-ha trapping plots (Additional file 2), whereas by the end of the cycle in spring 2009 there was only one plot with infected bank voles (Fig. 2).

**Fig. 4:**
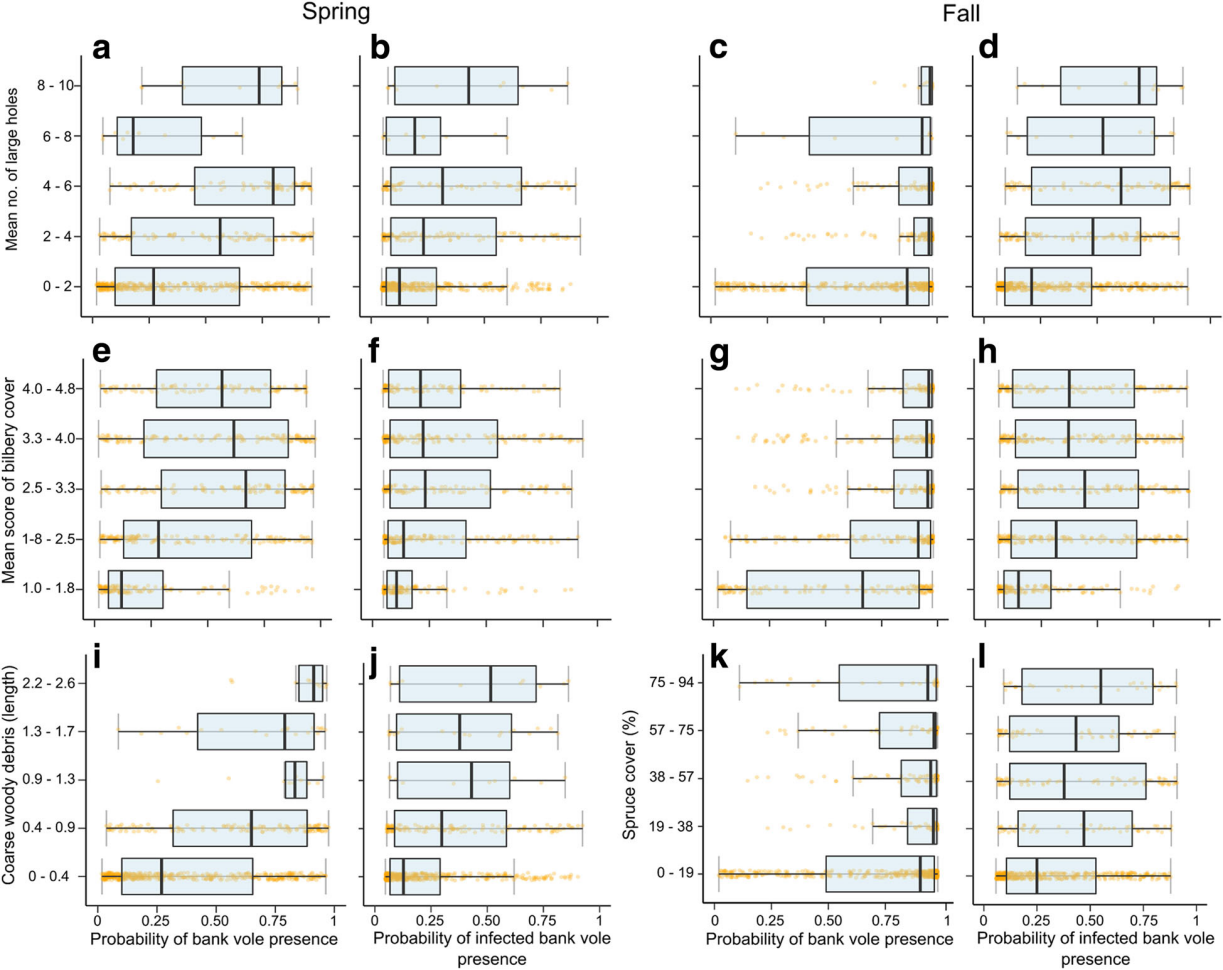
Predicted relationships between mean micro-habitat variables and presence of all bank voles (**a**, **c**, **e**, **g**, **i**, **k**) and infected bank voles (**b**, **d**, **f**, **h**, **j**, **l**) in spring (two left columns: **a**, **b**, **e**, **f**, **I**, **j**) and fall (two right columns: **c**, **d**, **g**, **h**, **k**, **l**) in fall 2003–2013. Large holes and bilberry cover were important predictors in both spring and fall models predicting overall bank vole and infected bank vole presence (**a**-**h**). In spring, coarse woody debris was an important predictor for both bank vole and infected bank vole presence (**i**, **j**). In fall, spruce cover (%) was an important predictor for the presence of infected bank voles (**l**) but not of overall bank vole presence (**k**). The boxes encompass percentiles: 25%–50% and the error bars represent the 95% confidence intervals

In the four models predicting overall bank vole and infected bank vole presence in spring and fall, micro-habitat variables related to availability of cover and food were important. All models performed well; AUC was ≥84 and 25–40% of the deviance was explained (Table 2). Among the minimum number of variables that cumulatively explained 85% of the deviance, ‘Bilberry’ and ‘Large holes’ were present in all four models (Table 3, Fig. 4a-h). The relative importance (%) of ‘Large holes’ was above 10% in three models out of four (Table 3). ‘Shrubs’ and ‘Spruce’ were present in three models out of four, while ‘CWD’ (coarse woody debris) was important for the two spring models (Fig. 4i, j). Further, ‘FWD’ (fine woody debris), ‘Tree layer 1’, ‘Tree layer 2’, ‘Uveg’ (umbrella vegetation), and ‘Lingonberry’ were present in two models.

**Table 2:** Performance of spring and fall models predicting the presence of any bank voles and infected bank voles in 58 1-ha plots in fall 2003–2013

Response	Spring		Fall	
	CV deviance explained (%)	AUC	CV deviance explained (%)	AUC
Bank vole presence	27	0.84	40	0.90
Infected bank vole presence	25	0.84	26	0.84

CV = cross-validated, AUC = area under curve (see Methods)

**Table 3:** Relative importance (%) of micro-habitat variables in the four models (two per season) predicting overall bank vole presence and infected (Inf.) bank vole presence in 58 1-ha plots between fall 2003 and 2013

	Relative importance (%)			
	Spring		Fall	
Habitat variable	Bank vole presence	Inf. bank vole presence	Bank vole presence	Inf. bank vole presence
Bilberry	**7.4**	4.0	**7.6**	3.6
Shrubs	4.0	**5.8**	4.7	-
Cobbles	-	-	-	3.0
CWD	**6.8**	4.3	-	-
FWD	3.2	-	-	**5.1**
Large holes	**6.8**	**10.3**	**11.4**	**12.1**
Lichens	-	-	2.6	-
Lingonberry	**8.1**	**4.8**	-	-
Pine	-	-	4.2	-
Spruce	4.1	3.9	-	3.7
Stoneholes	-	-	**4.9**	-
Tree layer 1	-	-	2.7	3.5
Tree layer 2	4.6	4.4	-	-
Uveg	-	-	3.2	**4.2**

The three variables with highest relative importance (%) in each model are given in bold

**Table 4:** Results from the validation of models predicting presences and absences of infected bank voles in an independent area (17 plots in 2007–2010 and 2015)

	Presence (TPR)	Absence (TNR)
Spring	41 / 43 (0.95)	21 / 42 (0.50)
Fall	49 / 54 (0.91)	24 / 31 (0.77)

The values represent the proportion of correctly identified positive (TPR) and negative (NPR) instances of infected bank vole presence in spring and fall

**Fig. 5:**
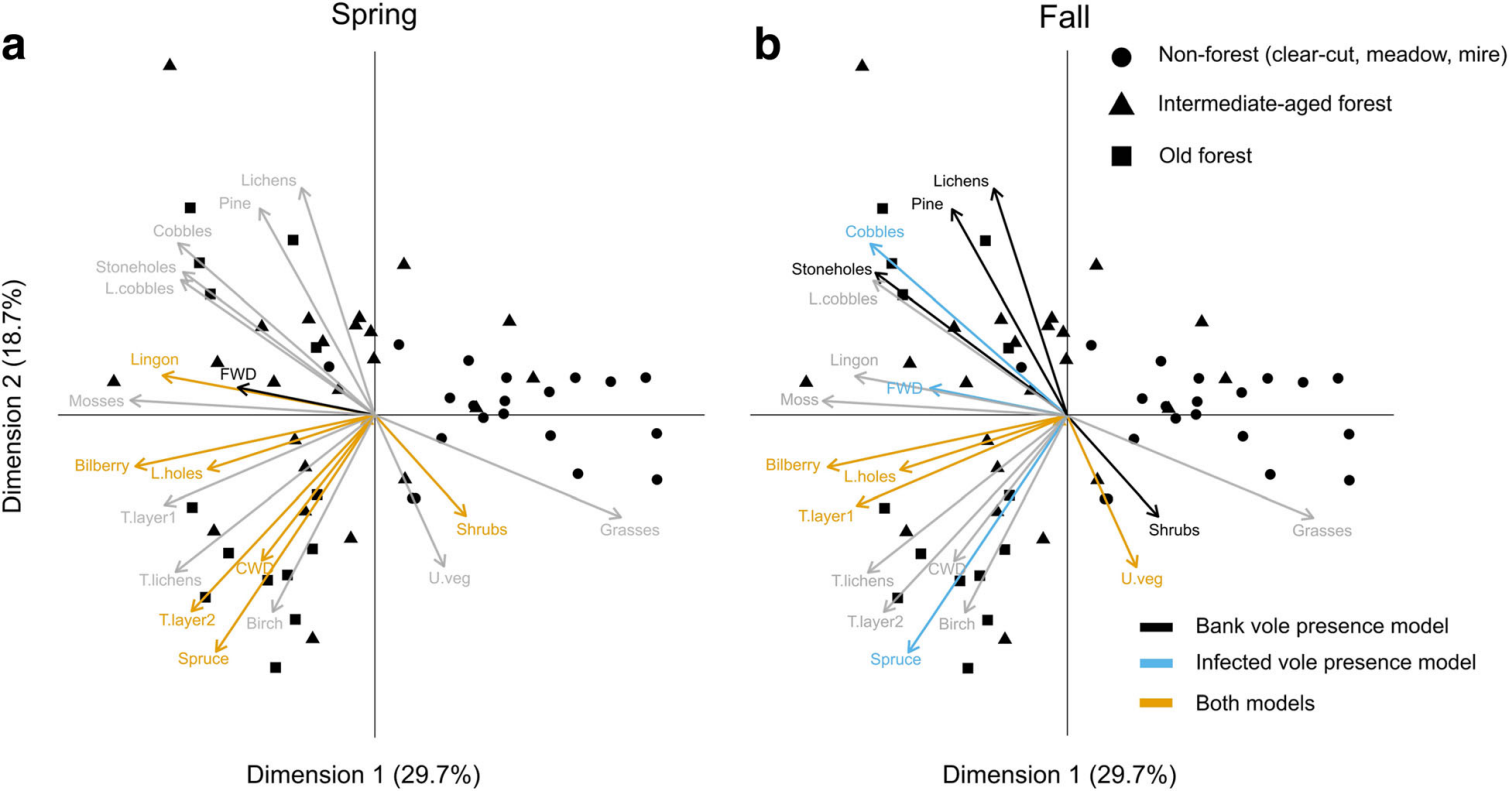
PCA of habitat variables and their relation to individual plots in different habitat types (58 1-ha pltos). Colours of the variables represent whether or not they were included in bank vole presence model, infected bank vole presence model, both, or neither (*grey*) in (**a**) spring and (**b**) fall

To contextualize habitat variables that were important for predicting overall bank vole and infected bank vole presence, we overlaid the results of the spring and fall models on a PCA bi-plot defined by the trapping plots and micro-habitat variables (Fig. 5). Plots in old forests and in non-forests were clearly separated by the PCA, whereas plots in intermediate-aged forests were spread along the environmental gradients, overlapping with plots in both old forests and clear-cuts and meadows.

In spring, the models predicting overall bank vole presence and infected bank vole presence were similar and shared seven out of the eight most important micro-habitat variables, e.g. ‘CWD’ (Table 3, Fig. 4i, j, 5a). The presence of bank voles in general and infected bank voles was predicted by micro-habitat variables typical of spruce forests, and variables related to cover and food availability, such as ‘Coarse woody debris’, ‘Bilberry’, and ‘Lingonberry’, were important. However, fall models diverged and only shared four variables out of ten (Fig. 5b). For example, ‘Spruce’ was not an important predictor of overall bank vole presence in fall (Fig. 4k, l), but was important for the presence of infected bank voles, the latter more likely to occur in plots rich in cover such as ‘Large holes’, ‘FWD’, and ‘Uveg’ (umbrella vegetation) (Table 3).

Both spring and fall models predicted the presence of infected bank voles in an independent area well (total number of predictions was 17 1-ha plots × 5 years = 85 instances). Model performance was fair in spring (AUC = 74) and good in fall (AUC = 83) [55] (Table 4). TPR was 0.95 and 0.91 in spring and fall, respectively. TNR was 0.50 and 0.77 in spring and fall, respectively. Hence in both seasons, the models predicted the presence of infected bank voles well, but performed worse in predicting absences, especially in spring (Table 4).

## Discussion

Predicting zoonotic risk involves identifying spatial determinants of host species and pathogen presence, especially in a heterogeneous landscape modified by humans. Bank voles are present in a variety of habitats in Fennoscandia and tolerate anthropogenic disturbance [23]. Their landscape distribution expands and contracts following the phases of the 3–4 year population cycle [35]. Using boosted regression trees, we showed that the presence of PUUV-infected voles can be successfully explained by micro-habitat properties and extrapolated to an independent area. According to our knowledge, this is the first study that utilizes boosted regression trees to predict and then validate zoonotic hazard. We found that during spring, variables related to the availability of cover and food in spruce forests predicted both overall bank vole and infected bank vole presence. In fall, the presence of PUUV-infected voles was more likely in habitats where cover was abundant, despite the broad bank vole landscape distribution.

Bank vole presence in the landscape varied seasonally, due to summer reproduction followed by winter decline, and inter-annually depending on the phase of the population cycle. When host distribution declined in winter and during low-density years, PUUV-infected voles were frequently found in a few focal patches (Fig. 2, Additional file 2). These habitats functioned as infection “refugia” from which future colonization of the landscape may occur [6, 10, 49]. However, no plot harbored infected bank voles throughout the 10-year study period (Figs. 1, 3), and PUUV-infected voles were trapped in different plots during lower density phases (“increase” and “decline”) of different cycles (Fig. 3, Additional file 2). This suggests that although some plots promoted persistence of infected voles during adverse periods, there remains an element of stochasticity in the occurrence of infected voles at plot level.

In fall, bank voles were broadly distributed in the landscape (Fig. 1b). In spring after winter decline, bank voles were trapped frequently in old spruce forests characterized by availability of micro-habitat structures that provide cover (e.g. fine and coarse woody debris, large holes, and shrubs) and food (e.g. lingonberry and blueberry) (Fig. 4, Table 3). Similarly, Ecke et al. [23] found that although bank vole densities were high in clear-cuts and young forests, their over-winter survival was lower than that in old forests. In Belgium, bank voles were also found in preferred habitats with dense cover during low density years [56]. In the U.S., hantavirus hosts survived in habitats with more cover where predation risk was hypothesized to be lower [57].

The likelihood of infected vole presence in a given plot appeared sensitive to temporal changes in micro-habitat properties. Compared to an earlier study in the same area on PUUV spatial dynamics between 1979 and 1986 [6], PUUV-status of several plots changed (see results for specific examples). Detailed habitat data between 1979 and 1986 was not available, but clear-cutting and forest succession led to habitat changes between 1986 and 2003. The corresponding change in PUUV-status of several plots increases our confidence in the importance of micro-habitat variables in determining infection presence.

For horizontally transmitted zoonoses, host presence is a necessary but not sufficient prerequisite for pathogen presence. In spring, micro-habitat variables promoting bank vole presence almost perfectly predicted the presence of PUUV-infected bank voles. In plots where bank voles survived winter and were subsequently trapped, there was a high likelihood that they would be PUUV-positive. This may be related to higher PUUV-prevalence in spring in over-wintered voles compared to fall [18, 36]. Whereas in fall, despite broader overall bank vole distribution in the landscape in fall (Fig. 5b) [6], the presence of PUUV infected voles was delimited by micro-habitat variables related especially to cover and typical of old spruce forests (see contrast between Fig. 5a and b).

The contrast between predictors of landscape occurrence of bank voles and infected bank voles in fall provides an opportunity to explore potential differences between host and virus ecology, compared to an earlier study that was limited to one fall season [28]. We suspect that habitats with abundant cover can enhance virus survival outside the host by maintaining moisture and reducing penetration of UV radiation [32, 33]. Additionally, bank voles may survive longer in plots where cover is abundant and predation rates are likely lower. Large holes found under cobbles, logs, and stumps were the most important predictor of the presence of PUUV-infected voles in fall (Table 3, Fig. 4d). Bank voles may use these holes as nesting sites or corridors leading to higher rates of encounter among infected and susceptible individuals and possibly higher exposure to environmental PUUV. We hypothesize that such naturally occurring holes function as “infection hubs”. Consequently, PUUV maintenance and transmission may be higher in moist and mesic spruce forests compared to drier habitats with less undergrowth or structures such as dry pine forests.

The discrepancy between bank vole and infected bank vole distribution may also be related to bank vole demography. Dispersing voles after summer reproduction may carry maternal antibodies and thus remain uninfected for a period of time [39]. Hence although we excluded bank voles that were likely to carry maternal antibodies, voles trapped in fall may have not had sufficient time to be exposed and infected with PUUV, which may introduce a lag between the presence of voles in general and the presence of infected voles.

In the same study area, interspecific competition reduced infection prevalence and density of infected voles through the dilution effect [36], whereby a reduction of host density or contact rates between host individuals due to the presence of a dominant co-occurring species ultimately reduces pathogen transmission [58]. The main forest competitor of bank voles, the grey-sided vole (*Myodes rufocanus*) prefers large holes under stones in pine forests [40]. Hence, after the dramatic decline of the grey-sided vole in the 1980’s and 1990’s [40], bank voles in spruce forests are undisputed in utilizing large holes, which reduces the likelihood of a dilution effect due to competition from grey-sided voles.

The importance of relatively wet and moist habitats for hantaviruses was previously pointed out in temperate Europe [59]. In Belgium, cover and resources, provided by beech trees, can predict the presence of infected bank voles [60]. In northern Sweden, core bank vole habitat is theoretically ideal for PUUV survival, namely mesic and moist forests rich in cover. In the U.S., persistently high risk of Sin Nombre hantavirus was also associated with moist habitats in deciduous or evergreen forests, compared to pastures and bare ground [10]. In Paraguay, animals infected with Jaborá hantavirus were more likely to be found where forest cover was thicker and moisture more likely retained. However, such habitat is less suitable for its host *Akodon montensis* [61], which was more abundant in human-disturbed habitats. The difference in habitat association between infected and non-infected *Akodon montensis* points to the importance of micro-habitat structure for viral survival and inter-specific encounter rate, and suggests a divergence between host and pathogen ecology in that case.

The models developed for the 58 1-ha plots around Umeå were able to predict presence of PUUV –infected bank voles in an area approx. 200 km north. The micro-habitat variables we measured were especially good at identifying plots with PUUV-infected bank voles, which makes it possible for practitioners and stakeholders to identify such places. Nevertheless, spring models overestimated presence of PUUV-infected voles (TNR = 0.50), which we propose was due to fewer positive plots in both areas in spring compared to fall. This may have resulted in the training data lacking sufficient number of positive plots to produce a model better to discriminate negatives from false positives in the validation area.

We attempted to maintain a balance between the feasibility of data collection for the models and the generality of their predictions on one hand, and explanatory power and interpretation on the other. For example, given the variables included here, practitioners do not need to trap bank voles to assess likelihood of presence of infected bank voles; although bank vole density would explain a large portion of the variation. Also, human disease risk is expected to be more closely related to the number of infected bank voles and human exposure to PUUV [1, 19, 62], rather than only the presence of infected voles. While evaluating the probability of finding infected bank voles is necessary for risk assessment, factors influencing human exposure to PUUV are also important [62]. For example, during winter 1990 in Germany, 15 out of 117 (8%) American soldiers camping on a bank vole infested terrain fell ill with PUUV infection, while not a single civilian case was registered in the region during that period. Soldiers who fell ill were more likely to have sighted rodents or slept on hay, and were hence more exposed to PUUV compared to uninfected soldiers and civilians in the same region [63].

Moreover, Surrounding landscape structure and connectivity is important for host movement and thus pathogen presence [6, 64], and we cannot rule out the possibility that bank voles moved into the plots just prior to trapping. Different years are characterized by different bank vole densities and landscape distribution [19]. Thus by including ‘Year’ as a predictor in all models, we attempted to account for landscape-scale processes, including bank vole influx into the trapping plots.

Near our study area, isolated patches of old forests are valued and maintained around urban and semi-urban houses. Given our results, we suspect that in such forest patches bank vole populations can persist and thus act as infection ‘refugia’ even when regional bank vole density declines. This is supported by earlier observations on human exposure to the virus, where most infections occur in or around human dwellings [62]. In the future, the connection between high-quality isolated forest patches and bank vole infestation of neighboring human dwellings ought to be explored.

## Conclusions

We demonstrated how micro-habitat variables can be used to predict presence of PUUV –infected hosts through boosted regression trees, whose predictive power is superior to traditional statistical models. We are unaware of previous studies on hantaviruses that validated habitat models and predicted infected host presence in independent data. In northern Sweden, moist and mesic old spruce forests, with abundance of structures that provide cover, e.g. large holes, and lingonberry and bilberry dwarf shrubs that provide both cover and food were most likely to harbor infected bank voles. For directly transmitted zoonoses, especially those carried by small mammals, similar predictive models based on habitat and micro-habitat variables in a well-studied area can contribute to rapid assessment of zoonotic risk in new locations in boreal landscapes. This negates the need for continuous sampling and processing of host individuals.

## Additional files

Additional file 1: Description of micro-habitat variables estimated in field and used in the statistical models. Data type: Text and table. (DOCX 37 kb)

Additional file 2: Landscape –scale presence of Puumala virus infected bank voles in 2003–2013 Data type: figure. (DOCX 323 kb)

## Abbreviation

PUUV: Puumala virus
