# Supplementary material for: Spatial prediction and validation of zoonotic hazard through micro-habitat properties: where does Puumala hantavirus hole – up?
Source: BMC Infect Dis. 2017 Jul 26;17:523. doi: 10.1186/s12879-017-2618-z (PMC5530527; doi:10.1186/s12879-017-2618-z)
Supplement: Supplementary file 1 — Description of micro-habitat variables estimated in field and used in the statistical models. Data type: Text and table. (DOCX 37 kb) [file 12879_2017_2618_MOESM1_ESM.docx]

**Additional file 1**

**Description of micro-habitat variables estimated in field and used in the statistical models**

Tl1: Upper tree layer on a 5 graded scale, at least 5 m high.

Tl2: Lower tree layer on a 5 graded scale. Tree layer should be at least 5 m lower in elevation than the upper tree layer.

Shrubs: Shrub layer on a 5 graded scale. The shrub layer should be at least 0.5 m high, but below 5 m in height.

Lholes (large holes): >5 cm diameter, number classes

Stoneholes (large holes under stones): >5 cm diameter, 6 number classes

FWD (Fine woody debris): Fine dead wood, diameter <10 cm on a 5 graded scale

CWD (Coarse woody debris): Coarse dead wood, total length estimated, to an accuracy of 0.5m

Cobbles: Stones >10 cm in diameter, on a 5-graded scale

Lcobbles (large cobbles): Stones >50 cm in diameter, on a 5 graded scale

Uveg (Umbrella vegetation): Vegetation cover >50 cm, on a 5 graded scale

Flveg (field layer vegetation): Vegetation in the field layer with height ≤50 cm

Grasses: Grass cover, 5 graded scale

Lichens: Cover of ground lichens, 5 graded scale

Bilberry: Cover of bilberry, on a 5 graded scale

Lingon: Cover of lingonberry, on a 5 graded scale

Mosses: cover of ground and stone growing mosses, on a 5 graded scale

Spruce: % cover

Pine: % cover

Birch: % cover

**5 graded scale:**

| % | Grade |
| --- | --- |
| 0 | 1 |
| 0 - 12 | 2 |
| 12 - 25 | 3 |
| 25 - 50 | 4 |
| >50 | 5 |

**6 number classes:**

| Number | Class |
| --- | --- |
| 0 | 0 |
| 1 – 4 | 1 |
| 5 – 9 | 2 |
| 10 – 19 | 3 |
| 20 – 39  ≥50 | 4  5 |
