# Supplementary material for: Spatial prediction and validation of zoonotic hazard through micro-habitat properties: where does Puumala hantavirus hole – up?
Source: BMC Infect Dis. 2017 Jul 26;17:523. doi: 10.1186/s12879-017-2618-z (PMC5530527; doi:10.1186/s12879-017-2618-z)

**Additional file 2**

**Landscape –scale presence of Puumala virus infected bank voles in 2003 – 2013**

Presence (red tiles) and absence (grey tiles) of Puumala virus - infected bank voles in 58 1-ha plots in spring and fall. Infection data was available between fall 2003 and 2013, and the different phases of the bank vole cycle are indicated. White tiles are plot where trapping of bank voles did not occur, as they overlapped water bodies.


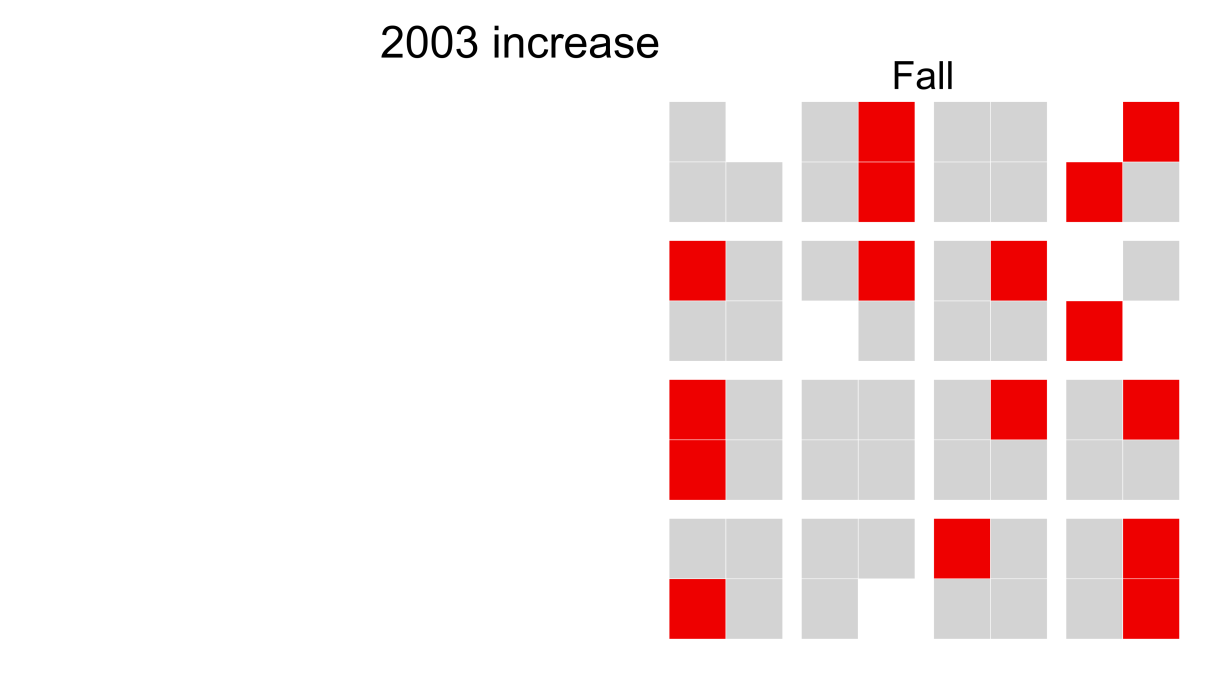


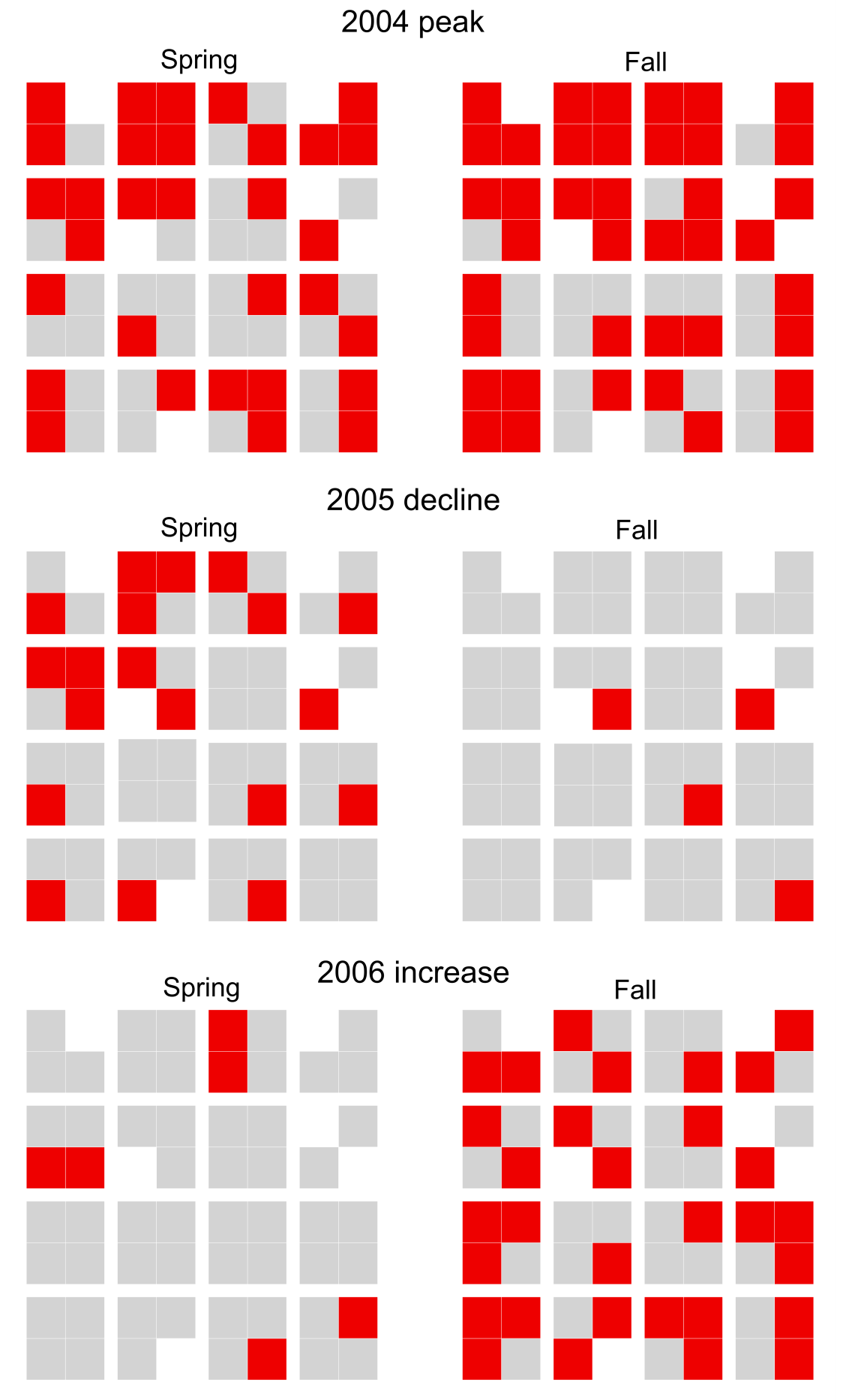


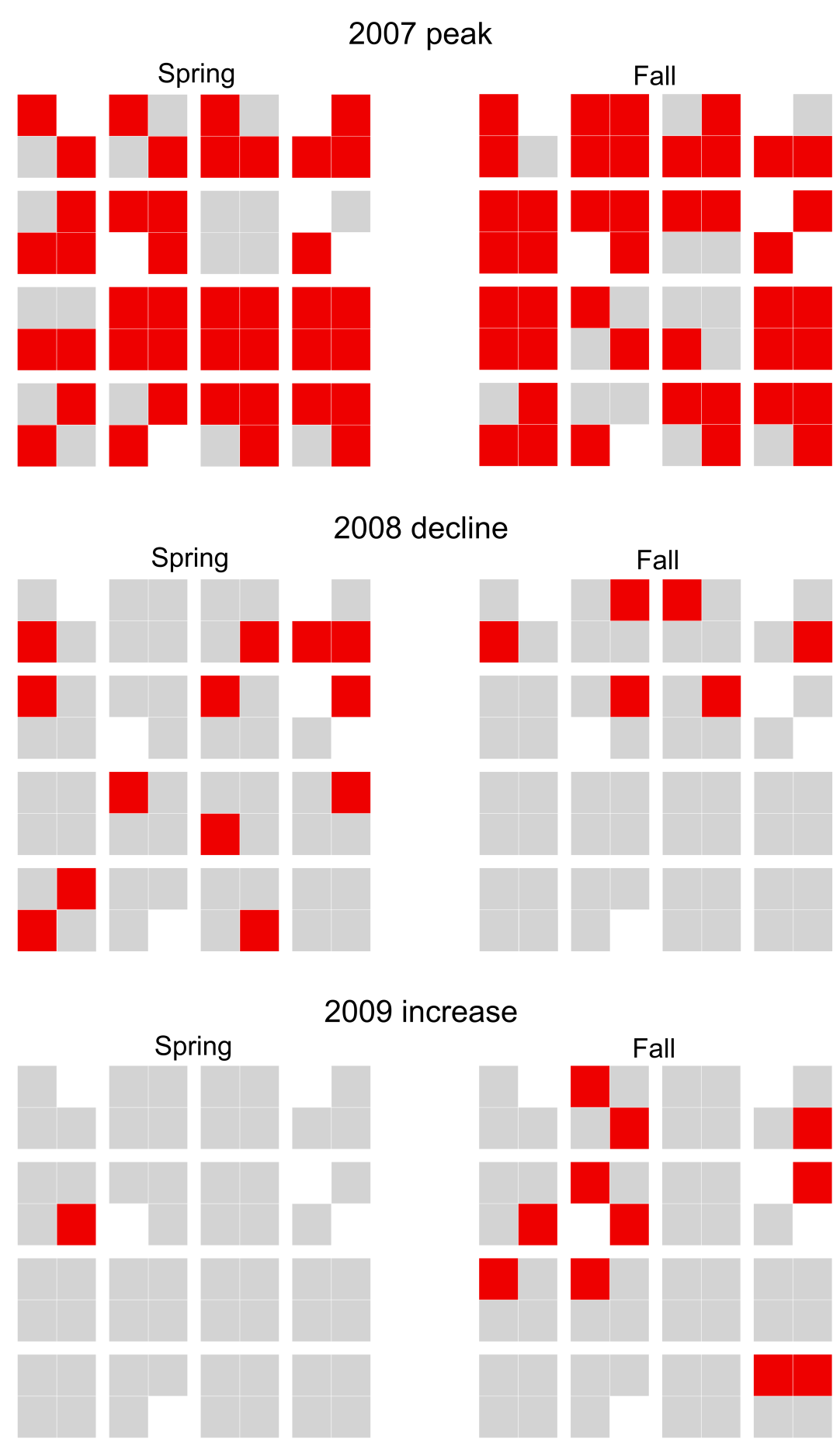


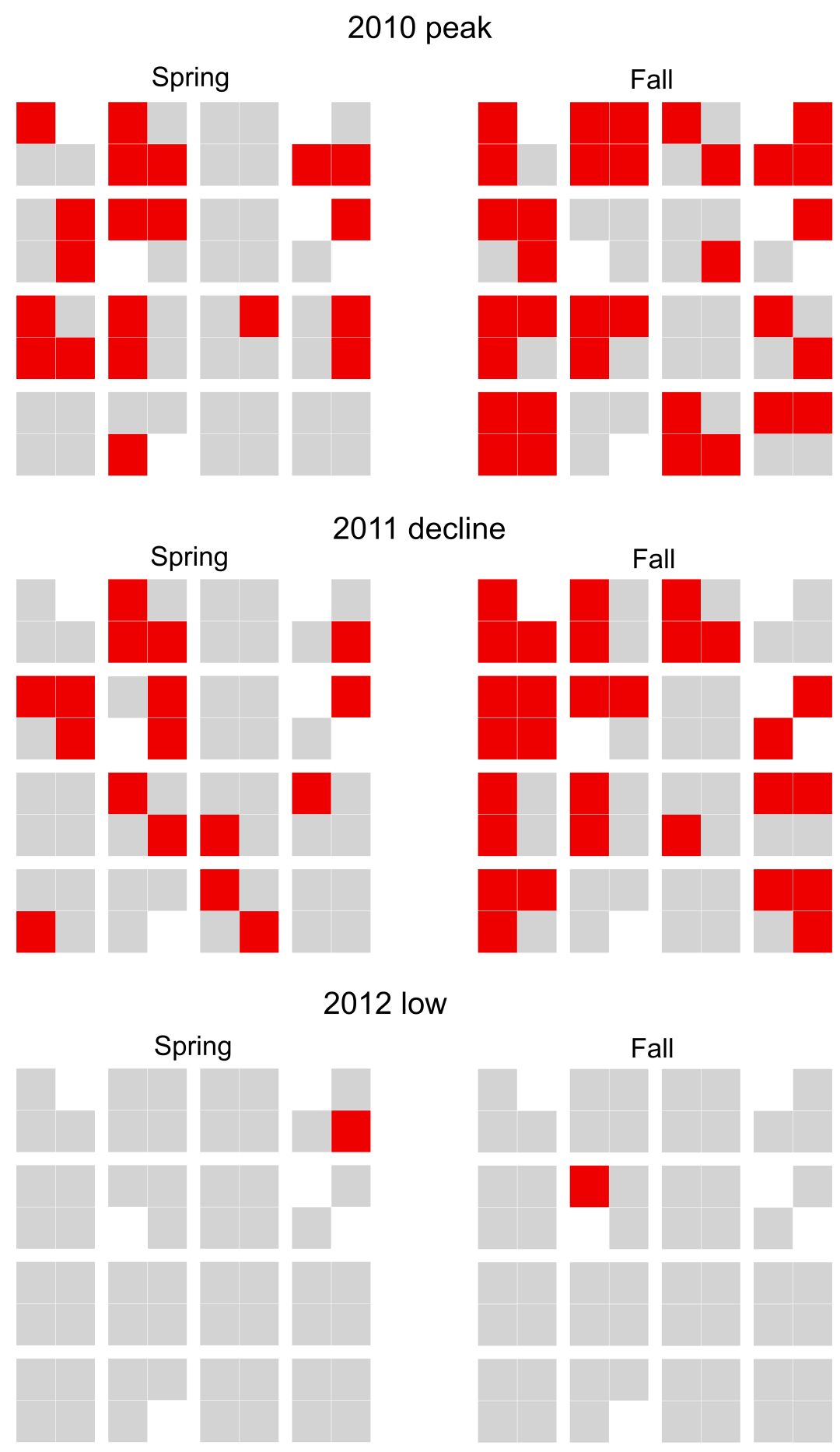


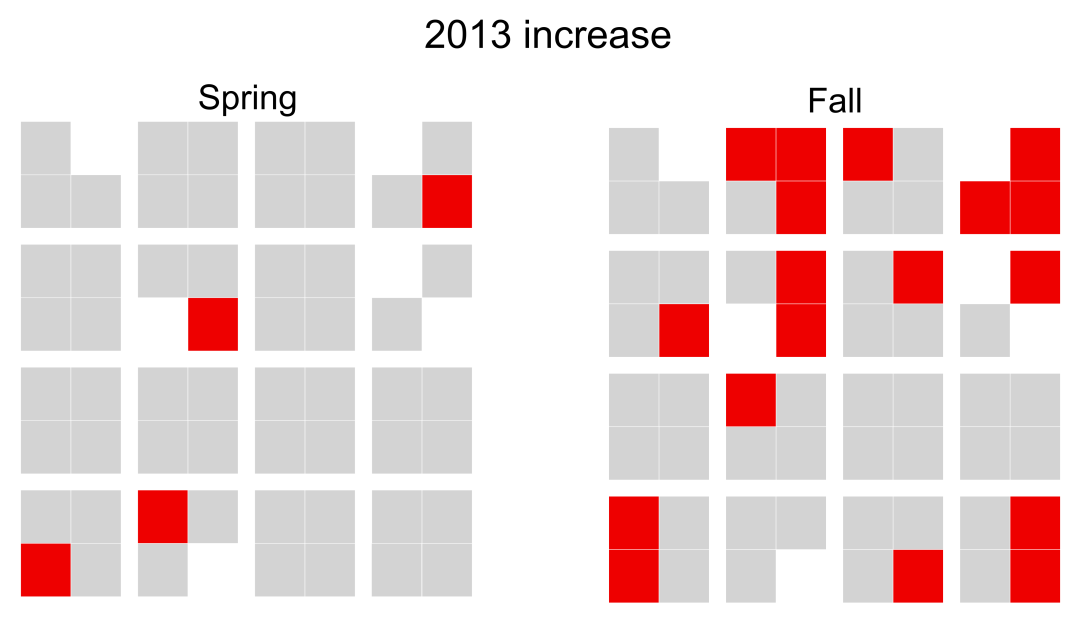

Supplement: Supplementary file 2 — Landscape –scale presence of Puumala virus infected bank voles in 2003–2013 Data type: figure. (DOCX 323 kb) [file 12879_2017_2618_MOESM2_ESM.docx]
